# The 22nd Chromatography Component of the *Fasciola gigantica* Excretory-Secretory Products Decreased the Proliferation of Peripheral Blood Mononuclear Cells from Buffalo

**DOI:** 10.3390/ani13040564

**Published:** 2023-02-06

**Authors:** Xiangxiang Yuan, Xiaoge Han, Xinping Kong, Linjing Hou, Kelong Wei, Mingtang Chen, Weiyu Zhang, Wenda Di

**Affiliations:** 1 College of Animal Science and Technology, Guangxi University, Nanning 530004, China; 2 Guangxi Colleges and Universities Key Laboratory of Prevention and Control for Animal Disease, Nanning 530004, China; 3 Guangxi Zhuang Autonomous Region Engineering Research Center of Veterinary Biologics, Nanning 530004, China; 4 Guangxi Key Laboratory of Animal Reproduction, Breeding and Disease Control, Nanning 530004, China; 5 Guangxi Buffalo Research Institute, Chinese Academy Agricultural Sciences, Nanning 530001, China

**Keywords:** *Fasciola gigantica*, Buffalo, cell proliferation, immunomodulatory, PBMCs

## Abstract

**Simple Summary:**

The 22nd chromatography component (F22) of the *Fasciola gigantica* excretory-secretory products (*Fg*ESP) shows better diagnostic value than the *Fg*ESP, but its immunomodulatory function and potential as a molecular vaccine candidate are unknown. Thus, the effect of F22 on the mitogen-induced proliferation of buffalo peripheral blood mononuclear cells (PBMCs) in the innate immune response was preliminarily studied, and its components were also explored. The results indicated that F22 significantly decreased the proliferation of PBMCs stimulated with mitogen. Two hundred and sixteen were identified as components of the F22, and these included eighty-six proteins present in more than one pathway and some with robust immunomodulatory ability. Further studies should be performed to investigate the immunomodulatory function of F22 in the adaptive immune response, and the components of F22 can be further studied as potential vaccine candidate molecules.

**Abstract:**

The 22nd chromatography component (F22) of the *Fasciola gigantica* excretory-secretory products (*Fg*ESP) shows better diagnostic value than the *Fg*ESP, and diagnostic methods based on F22 have also been established. Thus, exploring its immunomodulatory function and potential as a molecular vaccine candidate is attractive. In the present study, the effect of F22 on the mitogen-induced proliferation of buffalo peripheral blood mononuclear cells (PBMCs) in the innate immune response was preliminarily studied using the *Fg*ESP as a control. PBMCs were incubated with concanavalin A (ConA) and phytohemagglutinin (PHA) at optimal (1 µg/well) or suboptimal (0.25 µg/well) doses coupled with *Fg*ESP and F22 at different doses (1–16 µg/well). Cell proliferation was then assessed by microenzyme reaction colorimetry (3-(4,5-dimethylthiazol-2-yl)-2,5-diphenyl tetrazolium (MTT) assay). In addition, the components of F22 were also explored by mass spectrometry and then subjected to Kyoto Encyclopedia of Genes and Genomes (KEGG) analysis to infer their functions. The results indicated that *Fg*ESP decreased the proliferation of PBMCs stimulated with ConA and PHA at specific doses, whereas F22 significantly decreased the proliferation of PBMCs stimulated with ConA and PHA at both optimal and suboptimal doses (*p* < 0.05). Two hundred and sixteen proteins were identified in F22, and these included 86 proteins that could be assigned to more than one pathway and some with robust immunomodulatory ability. Further studies should be performed to investigate the immunomodulatory function of F22 in the adaptive immune response, and the components of F22 can be further studied as potential vaccine candidate molecules.

## 1. Introduction

Fascioliasis is a zoonotic disease caused by *Fasciola hepatica*, *Fasciola gigantica*, and their intermediate form that causes great global losses annually [1]. Drug deworming is a conventional method for preventing fascioliasis, but drug residues and drug resistance are less advantageous for its prevention [2]. Vaccines should be the primary method for preventing fascioliasis, and molecular vaccine candidates need to be screened based on an in-depth understanding of their immunomodulatory actions.

The interaction between *Fasciola* spp.—host manifests as the buffalo immune response, and *Fasciola* spp. immune evasion and excretory-secretory products (ESPs) play an important role in immune evasion by *Fasciola* spp. [3]. However, the complexity of ESPs has hindered explorations of their immunomodulatory activities, and subsequent research focused on specific proteins among ESPs has also yielded unsatisfactory results [4,5,6]. Encouragingly, a previous study revealed that the 22nd chromatography component of *Fg*ESP (F22) shows high diagnostic value, and an indirect ELISA and immunochromatographic strip based on F22 for bovine fascioliasis diagnosis has also been established and shows markedly better diagnostic effect than that of *Fg*ESP in terms of sensitivity and early diagnostic value [7,8]. Thus, determining whetherF22 has immunomodulatory activity would be interesting and helpful for vaccine candidate molecule screening.

Because the cell proliferation capacity reflects immune function [9], cell proliferation studies have been widely applied in immunomodulatory studies, such as traditional Chinese medicine research using mitogen-induced cells. A previous study [10] revealed that a *Centella asiatica* ethyl acetate extract could inhibit the proliferation of mouse spleen lymphocytes induced by mitogens, which indicates that the extract exerts a certain immunosuppressive effect. Another study [11] revealed that an extract of *Lepidium meyenii* could synergistically act with concanavalin-A (ConA) and lipopolysaccharide (LPS) to promote the proliferation of mouse spleen lymphocytes, indicating that the extract could enhance the immune activity of mouse spleen lymphocytes in vitro. Similarly, the present study was designed to explore the ability of F22 to regulate the proliferation of mitogen-treated peripheral blood mononuclear cells (PBMCs) from naïve buffalo.

## 2. Materials and Methods

### 2.1. Buffalo Maintenance

Four 6-month-old crossbred offspring of swamp buffaloes from Guangxi (China) and Murrah buffaloes, including males and females, were used in the present study. The buffaloes were born and maintained indoors, administered food and water ad libitum, and then used for subsequent PBMC collection only if they tested negative for fascioliasis by indirect ELISA based on F22 [7].

### 2.2. Preparation of FgESP and F22

*Fg*ESP was isolated as described previously [12]. In brief, adult *F. gigantica*, which were obtained from the gallbladder of naturally infected buffaloes at local abattoirs in Guangxi Zhuang Autonomous Region, PR China, were washed in phosphate-buffered saline (PBS, pH 7.2) and incubated in PBS for 3 h. The supernatants were then collected using a 20 μm nylon filter, centrifuged at 3000× *g* for 15 min, and sterilized by a 0.45 μm filter (Millipore, Boston, MA, USA). The protein concentration was determined using a bicinchoninic acid (BCA) Protein Assay Kit (TIANGEN BIOTECH, Beijing, China) and lyophilized for storage.

F22 was prepared according to a previous method [7]. Briefly, lyophilized *Fg*ESP was diluted with ddH_2_O, and the concentration was adjusted to 10 mg/mL. *Fg*ESP was then loaded onto a protein flash-chromatography system (GE, Boston, MA, USA) according to the corresponding instructions, 5 mL samples of the *Fg*ESP were loaded, 2 mL fractions were eluted using sterile PBS at a flow rate of 1 mL/min at room temperature, and the 22nd fraction was collected.

### 2.3. Isolation of Buffalo-Derived PBMCs

PBMCs were isolated as described previously [13]; whole blood was collected from the jugular vein and transferred to 50 mL sterile EDTA-K2 anticoagulated tubes. After centrifugation at 800× *g* for 20 min, the middle albuginea layer was diluted in RPMI 1640 medium (Gibco, Carlsbad, CA, USA). The diluted samples were gently mixed with isometric lymphocyte separation liquid (TBD, Tianjin, China) and centrifugation at 580× *g* for 20 min. The PBMCs in the middle white layer were collected and washed three times with PBS. The PBMCs were resuspended in culture medium (complete RPMI: RPMI 1640, 10% fetal bovine serum (Gibco, Carlsbad, CA, USA), 100 µg/mL streptomycin, and 100 U/mL penicillin (Gibco, Carlsbad, CA, USA).

### 2.4. Assessment of PBMC Proliferation

PBMCs (1 × 10^6^ cells/mL) from each buffalo were cultivated in triplicate in the absence or presence of the mitogens concanavalin-A (Con A) and phytohemagglutinin (PHA) (Sigma, USA) at optimal and suboptimal doses (1 and 0.25 µg/well for each mitogen) along with *Fg*ESP or F22 (1, 2, 4, 8, and 16 µg/well) in 96-well plates (Corning, Corning, NY, USA) in a total volume of 200 µL. PBMCs in the absence of *Fg*ESP, F22, and mitogen were also used as controls. The plates were incubated at 37 °C in a humidified atmosphere with 5% CO_2_ for 72 h, 10 µL of 3-(4,5-dimethylthiazol-2-yl)-2,5-diphenyl tetrazolium (MTT, Sigma, St. Louis, MO, USA) diluted to a concentration of 5 mg/mL in PBS was then added to each well, and the plates were incubated at 37 °C and 5% CO_2_ for 4 h. Then, 96-well cell culture plates were centrifuged (1000× *g* at 4 °C for 10 min), the supernatant was discarded, and 100 µL of DMSO DMSO (Solarbio, Beijing, China) was added to each well. After 10 min, the optical density at 570 nm (OD_570_) was measured using a microplate reader (Bio-Rad, Hercules, CA, USA).

### 2.5. Calculations and Statistics

The calculation and statistical analyses were performed as described previously [14]. For each buffalo, two stimulation indices (SI_S_) were calculated as follows:

$${\mathrm{SI}}_{1}=\frac{\mathrm{mean}\;{\mathrm{OD}}_{570}\;\mathrm{of}\;\mathrm{triplicate}\;\mathrm{test}\;\mathrm{cultures}}{\mathrm{mean}\;{\mathrm{OD}}_{570}\;\mathrm{of}\;\mathrm{triplicate}\;\mathrm{control}\;\mathrm{cultures}}$$

$${\mathrm{SI}}_{2}=\frac{\mathrm{mean}\;{\mathrm{OD}}_{570}\;\mathrm{of}\;\mathrm{triplicate}\;(\mathrm{mitogen}+\mathrm{Fg}\mathrm{ESP}\;\&\;F_{22}\;)\;\mathrm{cultures}}{\mathrm{mean}\;{\mathrm{OD}}_{570}\;\mathrm{of}\;\mathrm{triplicate}\;\mathrm{mitogen}\;\mathrm{cultures}}$$

The percent inhibition (% inhibition) of proliferation induced by the molecules was calculated as follows: % inhibition = 100 − (100 × SI_2_). The OD_570_ values of the samples treated with the mitogens and mitogens + tested molecules were compared by the nonparametric Wilcoxon test. The percent inhibition of proliferation by the molecules was compared by the nonparametric Mann–Whitney U test. Differences between tested groups were considered statistically significant if the *p*-value was ≤0.05, as indicated in the figures by letters (*). The experiment was performed four times.

### 2.6. Mass Spectrometry Analysis of FgESP and F22

*Fg*ESP and F22 were divided into tubes (100 μg/tube), and a Q Exactive chromatographic mass spectrometer was used to identify the protein components by mass spectrometry. *F. gigantica* (ST46835_*Fasciola gigantica*_13099.fasta) was selected for library comparison of the *Fg*ESP, whereas *F. hepatica* (ST_*Fasciola hepatica*_[6192]_15305.fasta) was selected for library comparison of F22. Proteins for which the number of unique peptides ≥ 2 were selected for subsequent analysis to detect the number of proteins related to *Fasciola* spp. Proteome Discoverer 1.3 (Thermo Scientific) software was used to match and score the original map files (raw files) from the peptide identification with the Q Exactive chromatographic mass spectrometer through SEQUEST software. According to the criterion FDR < 0.01, the peptides were screened based on the confidence, and the presence of at least two unique peptides was used for identification to obtain highly reliable qualitative results.

## 3. Results

### 3.1. Preparation of FgESP and Its Gel Filtration Chromatography Component F22

*Fg*ESP and F22 were collected as previously described [15]. The chromatogram of *Fg*ESP obtained by gel filtration chromatography showed four higher peaks of UV280 (P1, P2, P3, and P4), and F22 was located under the P1 peak (not shown). F22 obtained after elution was collected for concentration determination of the concentration, and F22 was then adjusted to specific concentration for further use.

### 3.2. FgESP Decreased Mitogen-Stimulated PBMC Proliferation at Specific Dose

**Figure 1:**
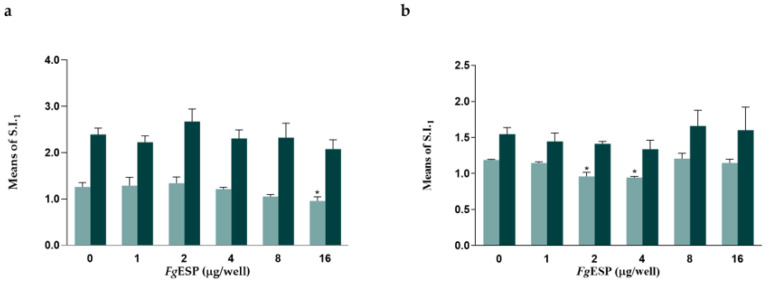
Regulatory effect of different concentrations of *Fg*ESP on the proliferation of buffalo PBMCs induced by 0.25 µg/well (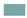) and 1 µg/well (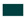) mitogen. (**a**). Regulatory effect of *Fg*ESP on ConA-induced PBMC proliferation. (**b**). Regulatory effect of *Fg*ESP on PHA-induced PBMC proliferation. The symbol (*) indicates significant difference (*p* < 0.05) between the ConA and ConA + *Fg*ESP groups or the PHA and PHA + *Fg*ESP groups at a suboptimal mitogen dose.

*Fg*ESP at multiple concentrations (1~8 µg/well) had no effect on the proliferation of PBMCs stimulated with ConA at either dose, whereas *Fg*ESP at 16 µg/well decreased the proliferation of PBMCs stimulated with ConA at a suboptimal dose (*p* < 0.05) (Figure 1a).

*Fg*ESP had no effect on the proliferation of PBMCs stimulated with PHA at the optimal dose. With the suboptimal dose of PHA, *Fg*ESP at 2 and 4 µg/well significantly decreased proliferation (*p* < 0.05) (Figure 1b). The percent inhibition induced by *Fg*ESP is shown in Table S1.

### 3.3. F22 Significantly Decreased Mitogen-Stimulated PBMC Proliferation

**Figure 2:**
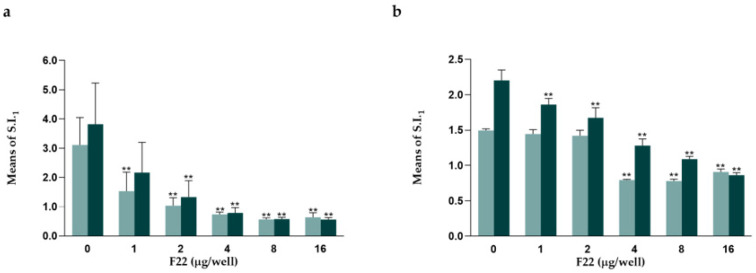
Regulatory effect of different concentrations of F22 on the proliferation of buffalo PBMCs induced by 0.25 µg/well (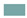) and 1 µg/well (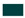) mitogen. (**a**). Regulatory effect of F22 on ConA-induced PBMC proliferation. (**b**). Regulatory effect of F22 on PHA-induced PBMC proliferation. The letters (**) indicate significant differences (*p* < 0.01) between the ConA and ConA + F22 groups or the PHA and PHA + F22 groups at 0.25 µg/well and 1 µg/well, respectively.

The ConA-induced proliferation of PBMCs was decreased by F22 (2~16 µg/well) (Figure 2a). With an optimal dose of ConA, F22 at multiple concentrations (2~16 µg/well) clearly decreased PBMC proliferation (*p* < 0.01). With a suboptimal dose of ConA, F22 at 1~16 µg/well decreased PBMC proliferation (*p* < 0.01).

The PHA-induced proliferation of PBMCs was also decreased by F22 (4~16 µg/well) (Figure 2b). With an optimal dose of PHA, F22 at multiple concentrations (1~16 µg/well) decreased PBMC proliferation (*p* < 0.01). With a suboptimal dose of PHA, F22 at 4~16 µg/well clearly decreased PBMC proliferation (*p* < 0.01), whereas at lower doses (1 and 2 µg/well), F22 had no regulatory effect on PBMC proliferation. The percent inhibition induced by F22 is shown in Table S2.

### 3.4. Analyses of the F22 Composition by Mass Spectrometry and KEGG Analyses of the Components

*Fg*ESP and F22 were subjected to mass spectrometry analysis (with a Q Exactive combined mass spectrometer), and proteins with ≥2 unique peptides were selected for analysis. *Fg*ESP contains 548 proteins, whereas F22 contained 216 proteins (Table S3). Various well-explored proteins, such as fatty acid binding protein (FABP) (*Fg*ESP score: 323.31), calcium-binding protein (CaBP) (*Fg*ESP score: 323.31), and heat-shock protein (HSP) (*Fg*ESP score: 323.31), were present in *Fg*ESP. Cathepsin L (CatL) (F22 score: 143.76), thioredoxin peroxidase (TPx) (F22 Score 103.37), and fibronectin type II domain (F22 Score 100.63) were present in F22 with high scores (Table S3).

**Figure 3:**
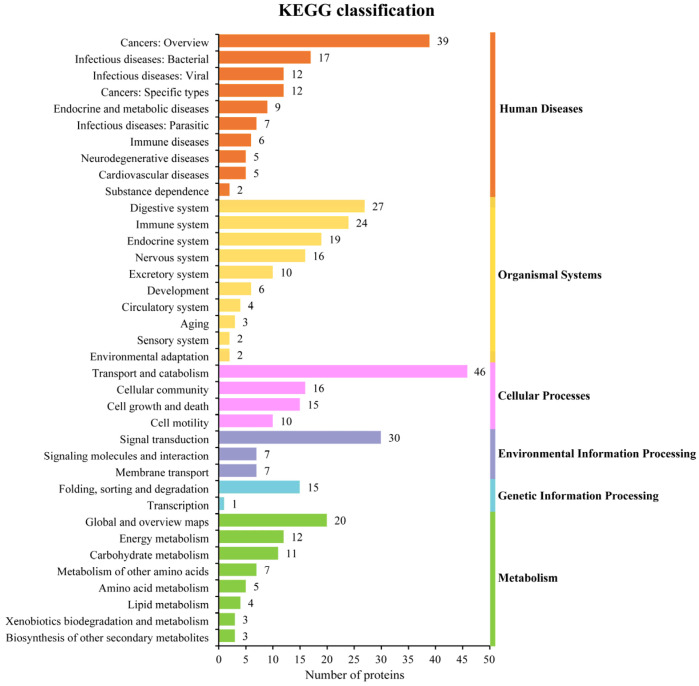
KEGG classification histogram of F22. Among 216 proteins, 125 proteins were clustered into specific pathways.

The KEGG pathways were screened to classify and analyze the components of F22. Among 216 proteins, 125 proteins were clustered into specific pathways. The most abundant pathways represented were “Human Diseases” and “Organismal Systems”, with 114 and 113 proteins, which accounted for 91.20% and 91.40% of all annotated proteins, respectively. Within “Human Diseases”, “Cancers: Overview” had the highest number of proteins (31.20% (39) of proteins), whereas “Digestive system” had the highest number of proteins within “Organismal Systems” (21.60% (27 of proteins) (Figure 3). Moreover, 86 of these 126 proteins are active in more than one pathway. Heat-shock protein 70 (B1NI98) was found in 10 pathways including “Transcription (Genetic Information Processing)”, “Signal transduction (Environmental Information Processing)”, “Transport and catabolism (Cellular Processes)”, “Aging (Organismal Systems)”, and “Infectious diseases: Parasitic (Human Diseases)”. Phosphotransferase (A0A2H1CVQ8) was present in eight pathways including “Biosynthesis of other secondary metabolites (Metabolism)”, “Signal transduction (Environmental Information Processing)”, “Endocrine system (Organismal Systems)”, and “Endocrine and metabolic diseases (Human Diseases)”; and 14-3-3 protein (A0A2H1CJE1) was involved in five pathways including “Signal transduction (Environmental Information Processing)”, “Cell growth and death (Cellular Processes)”, “Nervous system (Organismal Systems)”, and “Infectious diseases: Viral (Huma n Diseases)”. Glutathione S-transferase protein (A0A2H1C357) was present in three pathways including “Xenobiotics biodegradation and metabolism (Metabolism)”, “Metabolism of other amino acids (Metabolism)”, and “Cancers: Overview (Human Diseases)”; Kunitz (A0A0U5GJT7) existed in two pathways: “Nervous system (Organismal Systems)” and “Neurodegenerative diseases (Human Diseases)”. The results from the KEGG pathway analyses of the F22 component are listed in Table S4.

## 4. Discussion

As is well known, *Fg*ESP functions poly-directionally in *F. gigantica*—buffalo interaction and attracts a great deal of attention. However, its complex composition hindered relevant research to some extent. Encouragingly, the chromatography component of *Fg*ESP, F22, shows substantial diagnostic value and immunosuppressive ability, which indicates that chromatography may act as a tool in complex, mixed-component identification and can be applied to immunomodulatory molecular screening.

ConA and PHA are two mitogenic substances that can stimulate the proliferation and activation of T lymphocytes. These substances can activate different T-cell subpopulations, and activated T lymphocytes then function in the cellular immune response. In a previous study [14], ConA stimulated CD2+, CD4+ and CD8+ T lymphocytes, and its stimulatory effects were stronger on CD2+ and CD4+ T lymphocytes than on CD8+ T lymphocytes. PHA mainly acts on CD4-/CD8-/CD3+ T lymphocytes in PBMCs [16]. Various helminth-derived molecular are involved in host immunomodulation and exhibit immune-suppressive on PBMCs regulation, including proliferation inhibition, which suggests their potential as vaccine candidate molecules. Recombinant *F. gigantica* Ras-related protein Rab10 (r*Fg*Rab10) enhances the apoptosis and migration of PBMCs, promotes the phagocytic ability of monocytes, and significantly inhibits cell proliferation [17]. *Filarial nematodes* (*Onchocerca volvulus* and *Acanthocheilonema viteae*) cystatins suppress the proliferation of human PBMCs and murine spleen cells [18]. Arginine kinase from *Haemonchus contortus* (*Hc*-AK) decreased the proliferation and increases the apoptosis of goat PBMCs [19]. Recombinant *H. contortus* excretory-secretory 15-kDa protein induced decreased migration ability of PBMCs and stimulated PBMCs to increase NO production [20].

In the present study, *Fg*ESP decreased mitogen-stimulated PBMC proliferation at specific doses, which is consistent with the findings reported by Zhang [12]. However, considering the limited number of experimental animals included in the study, a larger scale of experimental animals should be explored. Based on the various molecules contained in *Fg*ESP, it can be inferred that *Fg*ESP may consist of inhibitory and activator components with immunomodulatory functions. Specific molecules in *Fg*ESP, such as *Fg*14-3-3e and *Fg*TPx, have been identified, and relevant research is underway [13,21]. Further research will uncover the functions of *Fg*ESP components in the immunomodulatory activity of *Fg*ESP. F22 decreased mitogen-induced PBMC proliferation stimulated by both ConA and PHA. As the results indicate, it can be inferred that the components of F22 may affect different T-cell subpopulations, and identifying the correlation between specific molecules in F22 and their target subpopulations will deepen our understanding of specific immunomodulatory processes.

As components of FgESP, the immunomodulatory roles of CaBP (*Fg*ESP Score: 323.31) and CaM (*Fg*ESP Score: 263.37) have been preliminarily explored. For CaBP2 and CaM2, a previous study showed that calmodulin-targeted drugs can impede the growth of *Schistosoma mansoni*, *Plasmodium falciparum*, and malarial parasites [22]. r*Fg*-CaBP4 protein can actively bind to the surface of goat PBMCs and regulate the immune response of PBMCs [23]. The immunomodulatory effect of *Fg*CaBP2 and *Fg*CaM2 may need to be explored further.

TPx and cathepsin L, which exhibited high scores in the mass spectrometry analysis of both *Fg*ESP and F22, have also been extensively explored, suggesting their nonnegligible immunomodulatory roles in *F. gigantica* and *F. hepatica* infection. A previous study [21] showed that *Fg*TPx significantly inhibits PBMC proliferation. Prowse et al. identified macromolecules of *Fh*ESP that are capable of suppressing mitogen-induced sheep whole-blood cellular (lymphocyte) proliferation in vitro; then, *Fh*ESP was fractionated, and a proliferation assay showed that the suppressive activity of *Fh*ESP is shown to correlate with the presence of Cat L proteases [24]. Recombinant Cat L has also been used for vaccine trials, and vaccine trials in sheep and cattle have shown that purified *Fh*Cat L1 and *Fh*Cat L2 show a protective range of 33% to 79% against *F. hepatica* infection [25].

F22 exhibits stronger immunosuppressive ability than the *Fg*ESP, which is helpful for the screening of potential vaccine candidate molecules. FND (F22 Score: 100.63), β-TUB (F22 Score: 26.29), and HDM-1 (F22 Score: 20.95) were identified in F22. Fibronectin type III domain-containing 5 (FNDC5) is a transmembrane glycoprotein that can be hydrolyzed and lysed to form irisin, which has many benefits in preventing human diseases [26]. FNDC5 supplementation may inhibit the LPS-induced polarization of M1 macrophages and inflammatory cytokines production [27]. β-TUB is a potential drug target, and subtle differences in β-TUB between the host and parasite are sufficient to provide selective toxicity for some agents [28]. HDM-1 can be used as an immunotherapy molecule to treat type 1 diabetes, as suggested previously [29].

KEGG analyses indicated that 86 of the proteins in F22 are active in more than one pathway. Heat-shock protein 70 (Hsp70) is a very conserved and prevalent protein [30], and the cellular responsiveness to stress is largely regulated by the chaperone function of heat-shock proteins, which protect cells from subsequent injury [31]. *Schistosoma mansoni* Hsp70 may be associated with regulated cecal invasion as well as cecal transformation [32,33]. The 14-3-3 protein belongs to a family of signal transducers involved in fundamental cellular processes including signal transduction, cell cycle control, apoptosis, and protein transport [34,35,36,37,38]. The r*Fg*14-3-3e protein, which inhibits the proliferation and migration of goat PBMCs, inhibits phagocytosis of monocytes and may play an important role in the interaction of *F. gigantica* with goat PBMCs [13]. Glutathione S-transferases (GSTs) belongs to an enzyme family that is involved in cellular detoxification processes and mainly acts to catalyze the binding of glutathione to a variety of electrophilic toxic substrates, which makes the toxin more water soluble and more be easily cleared by the host [39], and subsequent studies have suggested its robust vaccine potential. Further studies should be performed to investigate the immunomodulatory function of the components of F22, which will facilitate the screening of potential vaccine candidate molecules.

## 5. Conclusions

In the present study, F22 decreased mitogen-induced naïve buffalo PBMC proliferation, indicating its strong immunosuppressive effect on the initial buffalo immune response. Further studies on F22 and its specific component-mediated immunomodulation under the adaptive immune state may facilitate the screening of potential vaccine candidate molecules for the prevention of fascioliasis.

## Data Availability

Not applicable.

## Supplementary Materials

The following supporting information can be downloaded at: https://www.mdpi.com/article/10.3390/ani13040564/s1, Table S1: The inhibition percentages of different concentrations of *Fg*ESP on buffalo PBMC proliferation induced by optimal and optimal doses of mitogen. Table S2: The inhibition percentages of different concentrations of F22 on buffalo PBMC proliferation induced by optimal and optimal doses of mitogen. Table S3: Comparative analysis of *Fasciola gigantica* ESP and F22. Table S4: KEGG pathway analyses of F22 component.
