# Supplementary material for: The 22nd Chromatography Component of the Fasciola gigantica Excretory-Secretory Products Decreased the Proliferation of Peripheral Blood Mononuclear Cells from Buffalo
Source: Animals (Basel). 2023 Feb 6;13(4):564. doi: 10.3390/ani13040564 (PMC9951737; doi:10.3390/ani13040564)
Supplement: Supplementary file 1 [file animals-13-00564-s001.zip › Table S1.pdf]

Table S1. The inhibition percentages of different concentrations of *Fg*ESP on buffalo PBMC proliferation induced by optimal and optimal doses of mitogen.

Mitogen	Dose (μg/well)	Concentration (μg/well)	Buffalo A (♂) Murrha	Buffalo B (♂) Murrha	Buffalo C (♀) Murrha	Buffalo D (♀) Native cross-bred
ConA	0.25	0	0.00	0.00	0.00	0.00
		1	1.96	6.69	-6.64	-9.80
		2	-33.69	-0.75	-2.26	8.35
		4	-9.28	2.64	9.84	9.82
		8	6.96	11.81	23.63	22.38
		16	9.28	17.21	34.09	33.40
	1	0	0.00	0.00	0.00	0.00
		1	4.66	-3.51	19.97	5.78
		2	-1.30	-14.02	0.30	-33.99
		4	-3.48	-1.11	20.42	-2.97
		8	6.25	2.21	21.48	-20.79
		16	1.68	13.65	22.69	14.19
PHA	0.25	0	0.00	0.00	0.00	0.00
		1	2.60	5.52	4.72	1.53
		2	22.51	22.73	20.50	11.48
		4	22.15	20.14	21.73	18.38
		8	-4.04	-8.38	3.82	2.52
		16	-0.48	1.25	8.24	5.11
	1	0	0.00	0.00	0.00	0.00
		1	8.59	4.41	20.65	-8.62
		2	8.59	6.27	14.02	5.56
		4	16.72	7.46	28.81	-0.73
		8	-14.69	-26.27	8.06	2.70
		16	-17.03	-23.73	29.02	-4.96
