# Supplementary material for: The 22nd Chromatography Component of the Fasciola gigantica Excretory-Secretory Products Decreased the Proliferation of Peripheral Blood Mononuclear Cells from Buffalo
Source: Animals (Basel). 2023 Feb 6;13(4):564. doi: 10.3390/ani13040564 (PMC9951737; doi:10.3390/ani13040564)
Supplement: Supplementary file 1 [file animals-13-00564-s001.zip › Table S2.pdf]

Table S2. The inhibition percentages of different concentrations of F22 on buffalo PBMC proliferation induced by optimal and optimal doses of mitogen.

Mitogen	Dose (μg/well)	Concentration (μg/well)	Buffalo A (♂) Murrha	Buffalo B (♂) Murrha	Buffalo C (♀) Murrha	Buffalo D (♀) Native cross-bred
ConA	0.25	0	0.00	0.00	0.00	0.00
		1	52.22	34.87	59.43	58.96
		2	66.55	61.26	58.08	77.54
		4	73.66	76.42	68.85	82.82
		8	80.01	84.79	78.95	82.08
		16	71.23	86.17	70.24	85.56
	1	0	0.00	0.00	0.00	0.00
		1	21.36	34.14	50.51	62.28
		2	55.61	68.32	74.89	60.80
		4	74.50	81.47	79.88	79.89
		8	81.73	87.69	83.51	84.58
		16	82.01	88.88	78.79	88.45
PHA	0.25	0	0.00	0.00	0.00	0.00
		1	2.34	-5.81	-0.29	15.62
		2	-1.17	-1.84	4.71	17.26
		4	46.20	36.70	47.06	56.49
		8	42.92	46.79	47.56	53.64
		16	40.35	34.71	35.29	46.28
	1	0	0.00	0.00	0.00	0.00
		1	24.23	21.78	11.67	2.20
		2	35.50	28.77	8.02	21.43
		4	44.03	43.35	38.37	41.01
		8	53.93	55.90	50.35	40.69
		16	65.19	63.03	54.30	59.94
